# Enhancing Cybersecurity Investment with FAIR-ROSI: A Responsible Cybersecurity Approach to Digital Society

**DOI:** 10.1007/s10796-025-10625-y

**Published:** 2025-07-08

**Authors:** Ying He, Tong Xin, Cunjin Luo

**Affiliations:** 1 School of Electronic Engineering and Computer Science, Queen Mary University of London, 327 Mile End Rd Bethnal Green, London, E1 4NS UK; 2 School of Computer Science and Electronic Engineering, University of Essex, Wivenhoe Park, Colchester, CO4 3SQ UK

**Keywords:** Risk Assessment, Return on Security Investment (ROSI), Factor Analysis of Information Risk (FAIR), FAIR-ROSI Model, Cybersecurity Qualitative Metrics, Cybersecurity Quantitative Metrics

## Abstract

Investment in cybersecurity is critical to protect information system security, preserve organizational interests, and fulfil social responsibilities. However, due to the lack of a transparent process, investors often struggle to assess the effectiveness of their investments. Traditional return on security investment (ROSI) can be considered as an economic indicator which reflects investment efficiency, but it often emphasizes investment costs and anticipated returns while overlooks cybersecurity related metrics. This paper proposes the FAIR-ROSI model that integrates five qualitative and quantitative cybersecurity metrics with the Factor Analysis of Information Risk (FAIR) model. It combines practical qualitative and quantitative indicators to enhance the granularity of the traditional ROSI model. We then use a case study to evaluate the FAIR-ROSI model. The results from pre and post control measures shows a narrow margin between actual and projected loss values and a significantly higher ROI compared to the total security expenditure.

## Introduction

Information systems security involves not only the protection of an organization’s sensitive data and assets, but also the obligation to maintain user privacy and protect the trust of stakeholders (Huang & Wang, 2021; O’Halloran & Griffin, 2019; Walton et al., 2021). Cybersecurity investment is a strategic decision of an organization (Culnan & Williams, 2009), which covers two dimensions: organizational interests and social responsibility (Fleischman et al., 2023). From the perspective of organizational interests, cybersecurity investment is a crucial measure for maintaining normal operations (Kosutic & Pigni, 2022; Shaikh & Siponen, 2024). For example, it includes protecting user data, mitigating financial disruptions, rectifying vulnerabilities, and maintaining the integrity of digital infrastructure. On the social responsibility front, consistent and sustainable cyber security investments ensure that organizations comply with local and international policies and regulations (Fielder et al., 2016; Jeong et al., 2019; Zamani et al., 2020), and fulfil their responsibilities to users, partners, and society, which is the duty of businesses as responsible participants in the digital economy (Pappas et al., 2023).

Nonetheless, underinvestment in cybersecurity persists as a pressing issue (Blau, 2017; Gordon et al., 2015). Investment in cybersecurity is usually lacking mainly because it does not directly generate revenue (Fedele & Roner, 2022; Gordon et al., 2018; Lee, 2021); instead, they are often viewed as cost expenditures aimed at reducing losses from security incidents (He et al., 2022; Janicke et al., 2021; Smith et al., 2021). Furthermore, the lack of transparency in investment assessment process exacerbates the disconnect between perceived value and actual needs of cybersecurity investments.

From a responsible investment perspective, improving transparency in cybersecurity investment assessment is vital. Decision makers require a clear, evidence-based framework and objective indicators to assess investment efficiency (Beissel, 2016; Paul & Wang, 2019). However, not all cybersecurity investment decision-makers have expertise in cybersecurity, and therefore often rely on the experience of the Chief Information Security Officer (CISO) (Dor & Elovici, 2016; Moore et al., 2016). Such decisions based on subjective judgement and experience are prone to bias (Borrero & Henao, 2017; Busenitz & Barney, 1997). Traditional return on security investment models can be considered as an economic indicator which reflects investment efficiency, but it often emphasizes investment costs and anticipated returns while overlooks cybersecurity related metrics. For example, the European Network and Information Security Agency (ENISA) (2012) proposed the Return on Security Investment (ROSI) model, which provides an exhaustive quantitative risk assessment of the financial impact arising from security incidents that influence ROSI. While it has an economic lens, highlighting the importance of reputation and regulatory compliance, their ROSI model overlooks non-economic dimensions, which narrows its viewpoint (Abrahamsen et al., 2021). Furthermore, the ROSI model, has an imbalanced focus on quantitative metrics, lacking qualitative security metrics that yield significant influence over cybersecurity investment choices. Relying solely on ROSI can lead to an inadequate grasp of the complexities and challenges within an organization’s cybersecurity landscape. According to Kesswan & Kumar (2015), using the ROSI model and focusing solely on Single Loss Expectancy (SLE), Annual Rate of Occurrence (ARO), and Annual Loss Expectancy (ALE) does not provide a robust and comprehensive assessment. The ROSI model, while simpler and useful for risk assessment, covers only a portion of the risk landscape. It does not account for interactions between metrics or the influence of other threatening events in the organization, and it lacks the detailed information needed to quantify the likelihood of various risk events and losses (Yaqoob et al., 2019). In summary, the lack of transparency of the cybersecurity investment process makes it challenging for organizational investors and CISOs to make informed investment decisions.

This paper integrates the FAIR model and the ROSI model and maps the qualitative and quantitative indicators of cybersecurity into the integrated model, with the purpose of enhancing cybersecurity risk management capabilities and improving the transparency in cybersecurity investment decision-making. The FAIR model offers a methodology and framework for converting technical cyber security risks and countermeasures into potential economic losses and costs by breaking down risks into quantifiable factors. By mapping five qualitative and quantitative indicators to the key factors in the FAIR model, technical indicators can be converted into measurable loss figures, which allows for the calculation of the risk reduction achievable through specific countermeasures. This combination provides a systematic approach for risk assessment and improve the precision of cybersecurity investment decisions.

The rest of the paper is organised as follows. Section 2 is a literature review of the research background and key concepts. In Sect. 3, we present the FAIR-ROSI model and the proposed methodology in details. The FAIR-ROSI model is evaluated in Sect. 4 using a case study. Section 5 discusses research contributions and practical implications. Section 6 concludes this paper.

## Theoretical Background and Related Work

### Cybersecurity Investment and its Models

Cybersecurity investment decision-making is the process by which an organization evaluates, selects, and allocates resources to reduce security threats and protect assets through risk assessment and economic analysis (Beissel, 2016; Benaroch, 2018). Existing studies have proposed a variety of cybersecurity investment models. These models can be roughly divided into economic-driven models, risk-driven models, and mixed methods models which attempt to balance economic and cyber risk considerations.

Economic-driven models focus primarily on cost-benefit analysis. Due to the complexities of evaluating and quantifying myriad cyber risks, leveraging quantitative outcomes for investment decisions proves to be more convincing (He et al., 2023; Loft et al., 2021). Previous studies have used a variety of financial assessment tools and methods to assess losses, costs, and benefits. Gordon and Loeb (2002) proposed a pioneering economic model for optimal security investment that considers potential losses and vulnerabilities. The model shows that not all vulnerabilities are worth fully fixing, but there is a critical point of diminishing marginal benefits. More recently, Gordon et al. (2020) integrated cost-benefit analysis into the NIST cybersecurity framework to prioritize control measures based on marginal risk reduction. Tatsumi and Goto (2010) introduced real options to evaluate the flexibility of delaying investment under uncertainty. Among them, ROSI metric is one of the most widely used methods (Schatz & Bashroush, 2017). ROSI can be used to assess cybersecurity investments effectiveness by comparing the cost of security measures with the financial benefits derived from preventing potential losses (Enisa, 2012). Kesswan & Kumar (2015) demonstrated the use of cost-benefit analysis in cybersecurity, detailing the return on investment (ROI) calculation considering factors such as Single Loss Expectancy (SLE) and Annual Loss Expectancy (ALE) among others. However, these economic-driven models heavily rely on financial indicators, simplify technical complexity (Yaqoob et al., 2019), and ignore non-economic factors, such as compliance and reputation risks. It may lead to investment decisions being out of touch with actual risk scenarios (Schatz & Bashroush, 2017).

Risk-based models identify, assess, and prioritize cyber threats to guide investment decisions. For example, the risk assessment process of NIST SP 800 − 30 divides risk levels through a threat likelihood and impact matrix (Ross, 2012). Allodi and Massacci (2017) proposed a real-time risk assessment method combined with vulnerability scanning logs to dynamically adjust investment priorities by counting the frequency of non-targeted attacks. However, risk-driven models lack an economic perspective and cannot directly answer the question of “how much to invest” (Fielder et al., 2016).

### Cybersecurity Qualitative Metrics and Assessment

Catota et al. (2018) emphasized the significance of qualitative metrics such as threat responsiveness, but they also noted a lack of comprehensive analysis regarding critical infrastructures, especially in quantifying the impact of security responsiveness metrics on an organization’s security capabilities. Additionally, Naseer et al. (2021) discussed how cybersecurity incident response (CSIR) tends to be application-specific and categorized analytical insights into four types—real-time, forensic, predictive, and descriptive—essential for proactive CSIR and threat intelligence. Yet, they missed specifying crucial sub-indicators like response and recovery times in the CSIR process. Zadeh et al. (2020) detailed the prevalent cybersecurity threats facing organizations, classifying them into physical, human, communication and data, and operational threats. Their study emphasized the vulnerability of the IT and financial sectors to these threats, comparatively underlining the lesser impact of physical threats in these areas. Their work also incorporated threat categorization under frameworks like Microsoft’s STRIDE and NIST SP 800 − 30, aiding in the identification and application of qualitative metrics in this research.

Georgiadou et al. (2022) assessed the qualitative indicator of inherent threat and established a security culture framework with two levels (individual and organizational) across nine dimensions. The framework analyzed diverse domains to derive corresponding inherent threat factors. Given the subjective nature of human-induced intrinsic threat’s specific impact on qualitative indicators (threat level) and the challenge of precise quantification, this paper refrains from delving into the exact quantification of intrinsic threat. Nonetheless, Zadeh et al. (2020) comprehensively delineated the current cybersecurity threats organizations confront, categorizing them into four groups: physical threats, human threats, communication data threats and operational threats. The analysis highlighted the higher sensitivity of the IT and financial sectors to cybersecurity concerns, with physical threats being relatively less significant in these industries. The study provided a lens to emphasize the degree of cyber threats in the IT and financial sectors. It also depicted the four threat categories under Microsoft’s Threat Model (STRIDE) and the NIST SP 800 − 30 standard, contributing to this paper’s identification and reference of qualitative metrics.

Numerous organizations currently adopt a qualitative risk matrix for cybersecurity based on the NIST 800 − 30 global standard (Al Fikri et al., 2019; Ross, 2012). However, relying solely on such an approach essentially ties cyber risk assessment to professionals’ future predictions regarding specific attacks. Allodi and Massacci (2017) extend this perspective by introducing a quantitative assessment approach, evaluating the likelihood of non-targeted attacks through endpoint defence and periodic vulnerability assessment exercises. This method quantifies the likelihood of an attack more precisely. While this quantitative analysis offers a more scientific risk assessment method, concentrating solely on the number of vulnerabilities as a metric falls short in practice. Furthermore, Ghani et al. (2013) quantitatively evaluated software vulnerability qualitative indicators by leveraging the Common Vulnerability Scoring System (CVSS). This quantitative assessment methodology aids in prioritizing security investments, thereby minimizing losses from security vulnerabilities and optimizing the utility of security investments. The collective findings of these studies imply a need for introducing not only qualitative metrics analysis but also an emphasis on the impact of multiple quantitative metrics.

### Cybersecurity Quantitative Metrics and Assessment

As for the cybersecurity metrics, Ma (2021) combined qualitative and quantitative metrics to generate quantifiable data for structural comparisons. When assessing cyber vulnerability, it investigates the attack process, passive detection, and active detection. The paper introduces several common network vulnerability assessments, including qualitative and quantitative approaches. The assessment of system security risk emphasizes assets and threat rate, but the evaluation of threat loss remains insufficient. Regarding the specific analysis and calculation of quantitative metrics, Kim (2019) introduced a decision-making method for estimating malware risk indices. The study analyses the probability of malware and malicious activities (MAs) using a decision model that incorporates static and dynamic analyses to detect, identify, and classify various malicious activities and threat sources. By utilizing hierarchical analysis, it quantitatively assesses and quantifies the malware threat indices and subsequently examines the probability of malware and MAs. The primary emphasis is on quantitative metrics, probability of malware, probability of MAs.

Wang (2020) conducted an in-depth analysis of the vulnerability detection technique—the fuzzing technique. The findings from this study can provide improved detection tools for organizations using historical metrics statistics. CRISTEA (2021) introduced five security threats: ransomware, malware, advanced persistent threats (APT), third-party threats, and external actor sabotage. The study proposed a more practical risk management model primarily centred on analyzing the frequency of these five types of security events, commonly referred to as the frequency of the threat. Although Allodi and Massacci (2017) acquired a risk metric by adopting quantitative risk analysis in conjunction with traditional qualitative analysis, organizations seeking a more intuitive organizational risk value need to estimate threat probability and subsequently establish a targeted baseline based on the calculated risk value. In this context, a suitable approach to assess threat frequency involves initially comprehending the nature of the security event and then estimating the associated threat probability.

## Methodology

**Fig. 1:**
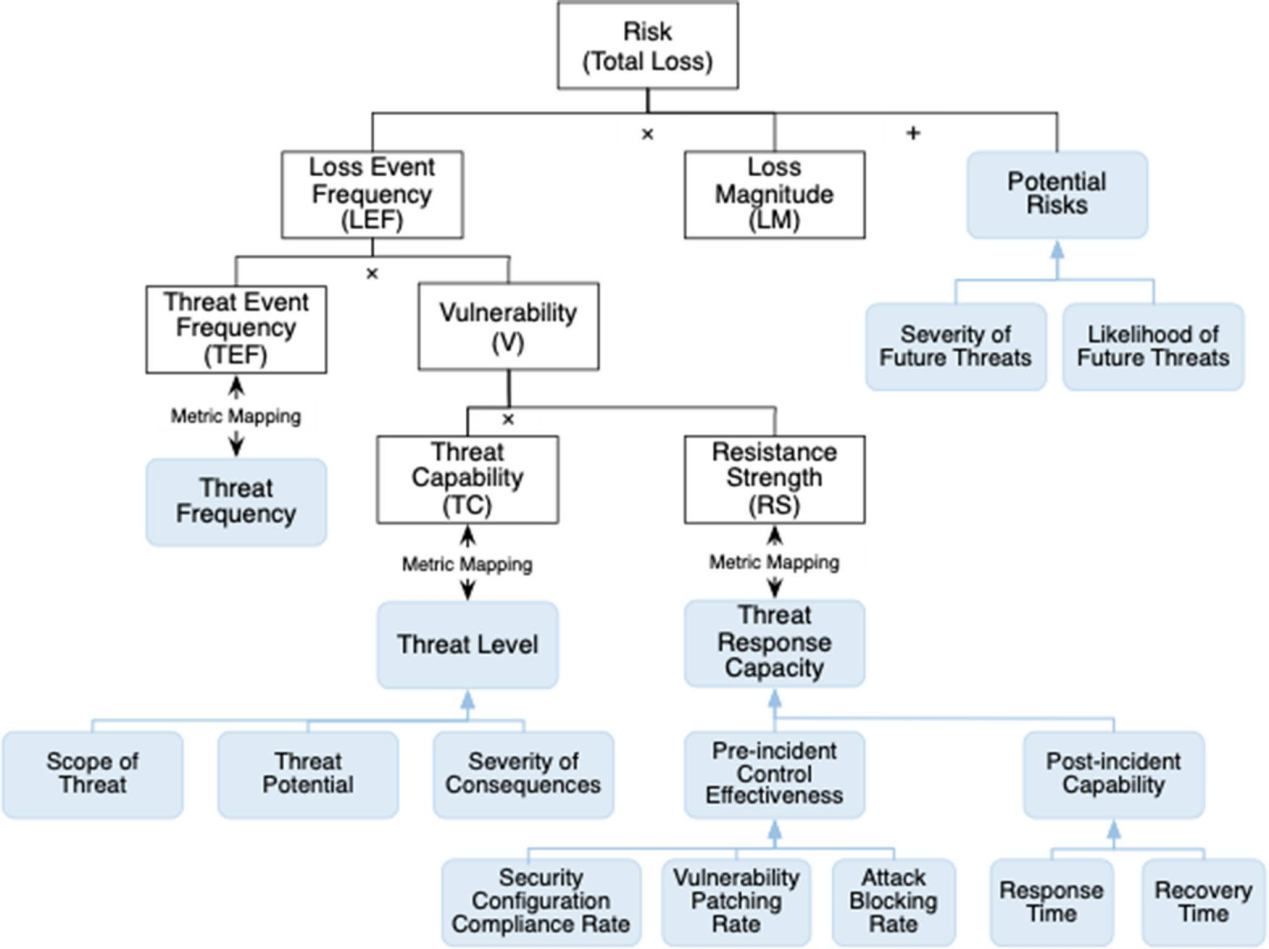
High level FAIR-ROSI model. The elements enclosed in black boxes represent components of the FAIR model. The blue boxes indicate the qualitative and quantitative metrics we have selected, along with their corresponding sub-metrics.

This section introduces the proposed FAIR-ROSI model. This model aims to evaluate the rationality of cyber security investment decisions through the results of ROSI. The calculation of ROSI is based on the components and quantification methods in the FAIR model. We choose a series of threat response properties, including threat level, threat frequency, threat response capability. We map these metrics and add a potential risk component to FAIR components. The metrics were selected based on three criteria informed by Ross (2025)’s research: relevance to the organizational risk assessment, consistent with existing cybersecurity frameworks, and can be quantified through existing organizational tools and processes. This mapping is inspired by (Qamar et al. 2017)’s work, we map the threat response properties to FAIR components that are semantically similar and comparable. The threat response properties serve as a bridge between evaluating FAIR components and calculating ROSI, as these are the key properties used by both FAIR and ROSI. In this section, we first introduce the components and quantification methods of the FAIR model, then explain how the qualitative and quantitative metrics of the threat response properties map to FAIR components, and finally introduce the baseline settings of the qualitative and quantitative metrics. Figure 1 shows the high-level proposed FAIR-ROSI model with specific qualitative and quantitative metrics elaborated in Sect. 3.2.

### The Components and the Quantification in the FAIR Model

As shown in Sect. 2.1 and Fig. 1, the FAIR model has eight key components (den Elzen & Lucas, 2005; Freund & Jones, 2014). Loss Event Frequency (LEF) refers to the frequency of loss events occurrence. LEF is affected by two sub-metrics: Threat Event Frequency (TEF) and Vulnerability (V). TEF refers to the likelihood of a threat event, while V denotes the susceptibility of an organization’s assets to a threat event. TEF can be further influenced by Contact Frequency (CF) and Probability of Action (PoA), and V is impacted by Resistance Strength (RS) and Threat Capability (TC), respectively. CF refers to the frequency at which a threat endeavors to exploit an asset, and PoA refers to the likelihood that a threat will execute a detrimental action after accessing an asset. RS assesses the effectiveness of organizations’ security controls, while TC reflects the capability of a threat. The formulas are outlined as follows:

1 $$\:\begin{array}{c}LEF=TEF\times\:V\end{array}$$

2 $$TEF=CF\times\:PoA$$

3 $$V=RS\times\:TC$$

Loss magnitude (LM) represents the magnitude of the loss expected at the time of the loss event. It comprises a primary loss magnitude (PLM) and a secondary loss magnitude (SLM). Among them, PLM is the loss directly caused by the loss event, and SLM is the further loss due to the primary loss. In this study, we will focus on the effect of PLM on FAIR modeling. The formula is outlined as follows:

4 $$LM=PLM\times\:SLM$$

Risk (R) is a combination of loss event frequency and loss magnitude. The formula is as follows:

5 $$R=LEF\times\:LM$$

### The Qualitative and Quantitative Metrics

This section introduces and expands the qualitative and quantitative metrics as shown in Fig. 1.

#### Qualitative Metrics

*Threat response capacity* assesses the level of response in the face of cyber threats and consists of two sub-metrics: *pre-incident control effectiveness* and *post-incident capability*. *Pre-incident control effectiveness* refers to the effectiveness of an organization’s control measures in identifying, preventing, and detecting threat events. According to the NIST Cybersecurity Framework (Ross, 2025), it can be measured via three observable metrics: the organization’s *security configuration compliance rate*, *vulnerability patching rate*, and *attack blocking rate*. The *security configuration compliance rate* measures the degree of compliance of the system’s security configuration with the security baseline specified by the organization, which can be assessed by the security configuration of the operating system and network devices. The *vulnerability patching rate* measures the organization’s efficiency to patch identified vulnerabilities in a certain period. This information can be obtained from organizations’ vulnerability scanning systems. The *attack blocking rate* is the percentage of threat attack attempts that an organization successfully blocks in a certain period, which can be obtained from SIEM and system protection logs. *Post-incident capability* is the organization’s ability to respond and recover after a threat incident occurs, which can be measured by response time and recovery time. *Response time* indicates the time required to identify and respond to a threat event; *recovery time*, which signifies the duration to restore normal operations. These sub-metrics together cover the before (i.e., control measures) and after (i.e., recovery time) stages of a cyber threat event, which assess the organization’s cybersecurity posture, making them a good measure of threat response capacity. *Threat response capacity* could be used to evaluate RS in FAIR model. It provides a more comprehensive view of an organization’s ability to respond to cyber threats, which not only considers RS, but also the speed of response and recovery, which are critical aspects of managing cyber threats.

*Threat level* refers to the specifics of cyber threats encountered by organizations. The specific quantification of the threat level is expanded to three sub-metrics: *threat potential* refers to how likely a specific threat is to occur; *severity of consequences* indicating the potential damage a threat could cause; and the *scope of threat*, which is the breadth of impact a threat could have. They collectively provide a detailed picture of the threat level. In FAIR-ROSI model, *threat level* could be mapped to TC. According to the definition of TC, threat potential can be considered as the likelihood of a threat agent applying force against an asset. The severity of consequences and scope of threat further detail the potential impact of this force, which is consistent with the FAIR model’s focus on quantifying risk. This model will quantify the likelihood, severity, and scope of impact using a 0–1 scoring system. Given that TC is subject to change, the quantification of threat level ensures that the organizations routinely reassess the magnitude of TC. This quantification approach aligns with the requisites of TC and contributes to increased operability and dynamic adaptability.

*Potential risk* is used to evaluate various future cyber threats an organization could face. The two sub-metrics are the *likelihood of future threats*, the *severity of future threats*. By incorporating potential risk, the FAIR model will be capable of accounting for risks that may emerge in the future. This model uses a 0–1 scoring system to quantify the likelihood and severity of the consequences of potential risks.

It is important to emphasize that, although these metrics are directly mapped to some components of the FAIR model and directly affect the evaluation of these components, they indirectly affect higher-level components through the inbuilt calculation models of the FAIR model. For example, the threat level directly affects our measurement of the threat capability and affects the evaluation of LEF through TC (i.e., threat level $\:\to\:$ TC $\:\to\:$ V $\:\to\:$ LEF). This design enables our model to assess the current security status and future risk situation more precisely.

#### Qualitative Metrics

*Cybersecurity return on investment (ROSI)* is an economic metric used to assess the effectiveness of an investment by measuring the return generated. ROSI is calculated by using Loss Event Frequency (LEF) and Loss Magnitude (LM) determined in the FAIR model, and investment guidance is provided based on the ROSI calculation results. A positive result indicates a favorable return on the investment, while a negative result suggests that the investment expectations have not been met.

*Threat frequency* is the actual number of times a specific threat event occurs within a designated time frame. Given that threat frequency relies on observed actual data for determination and can be tracked and updated through logs, monitoring systems and other data sources, mapping threat frequency to TEF allows the abstract concept to be translated into concrete observed threat frequency data.

### Baseline Setting

Each organization establishes its specific acceptable values for the metrics based on its cyber security environment and security capacity. These values represent the organization’s “normal” state for cyber performance metrics, and any metric deviations from this baseline, particularly those falling below it, can be deemed anomalies or risks. This model integrates the principles of NIST Special Publication 800 − 53 Rev. 4 (FORCE, 2013) to determine high and medium-low impact security systems. It primarily centers on the cybersecurity investment objectives of the organization and uses the historical security data of each organization as a foundation. The analysis is based on the average value of each metric across organizations.

#### Baseline Setting for Qualitative Metrics

To establish the value for *threat response capacity*, the assumption is that organizations can automatically compute values of the sub-metrics, *response time*,* recovery time*, and *effectiveness of control measures*. This computation can be accomplished by utilizing the log and event monitoring system, the security event management system (SIEM), and the fault ticketing system during the acquisition of threat responsiveness data.

The *threat response capacity* is determined based on the average of the *pre-incident control effectiveness* and *post-incident capability* metrics. *Pre-incident control effectiveness* can be assessed through three sub-metrics, *security configuration compliance rate*, *vulnerability patching rate*, and *attack blocking rate. Post-incident capability* is calculated based on response time and recovery time. The data can be sourced from the log and event monitoring system, the security event management system (SIEM), and the fault ticketing system, the security configuration of operating systems and network devices, etc.

The security configuration compliance rate (CCR) is calculated based on the ratio of the number of endpoints that meet the organization’s security requirements ($\:{EP}_{S}$) to the total number of endpoints ($\:{EP}_{T}$). The formula is as follows:

6 $$CCR=\raisebox{1ex}{${EP}_{S}$}\!\left/\:\!\raisebox{-1ex}{${EP}_{T}$}\right.\times\:100\%$$

The *Vulnerability Patching Rate (VPR)* is calculated based on the ratio of the number of fixed vulnerabilities ($\:{V}_{P}$) to the total number of critical vulnerabilities ($\:{V}_{T}$) within the organization:

7 $$VPR=\raisebox{1ex}{${V}_{P}$}\!\left/\:\!\raisebox{-1ex}{${V}_{T}$}\right.\times\:100\%$$

The *attack blocking rate* (*ABR*) is based on the ratio of the number of blocked attacks ($\:{A}_{B}$) to the total number of attacks detected ($\:{A}_{T}$):

8 $$ABR=\raisebox{1ex}{${A}_{B}$}\!\left/\:\!\raisebox{-1ex}{${A}_{T}$}\right.\times\:100\%$$

*Pre-incident control effectiveness* (*PCE*) is determined by the average of the above three indicators:

9 $$PCE=\raisebox{1ex}{$(CCR+VPR+ABR)$}\!\left/\:\!\raisebox{-1ex}{$3$}\right.$$

To calculate the *average response time (ART)* of the sub-metrics, let’s denote $\:{n}_{R}$ as the number of response times in the organization, and $\:{R}_{i}$ as the i^th^ response time. The formula for calculating ART is as follows:

10 $$ART=\left(\raisebox{1ex}{$1$}\!\left/\:\!\raisebox{-1ex}{${n}_{R}$}\right.\right)\times\:\sum\:{R}_{i}$$

Calculate the *mean recovery time (MRT)* of the sub-metric, where $\:{n}_{M}$ in the organization denotes the number of recovery times and $\:{M}_{i}$ denotes the i^th^ recovery time, then the MRT is:

11 $$MRT=\left(\raisebox{1ex}{$1$}\!\left/\:\!\raisebox{-1ex}{${n}_{M}$}\right.\right)\times\:\sum\:{M}_{i}$$

*Post-incident capability (PIC)* is determined by the average of the mean response time and the mean recovery time is:

12 $$PIC=\raisebox{1ex}{$(ART+MRT)$}\!\left/\:\!\raisebox{-1ex}{$2$}\right.$$

Thus, *threat response capacity (TRC)* is calculated as follows:

13 $$TRC=\raisebox{1ex}{$(PCE+PIC)$}\!\left/\:\!\raisebox{-1ex}{$2$}\right.$$

The qualitative metrics of *threat level (TL)* are assessed qualitatively based on *threat potential (TP)*,* severity of consequences (SC)*, and *scope of threat (ST)*, with assessment levels of low, medium, and high, corresponding to a specific range of values of 0–0.33, 0.34–0.66, and 0.67–1.0 (see Table 1).

**Table 1:** Qualitative threat level assessment form

Threat potential	Severity of consequences	Scope of threat	Numerical range
Highly unlikely	Minor impact	Affects a small part	0.0–0.33
Likely	Medium impact	Affects a part of the region	0.34–0.66
Highly likely	High impact	Affects entire organization	0.67–1.0

The three sub-metrics for measuring threat level require a coordinated effort from both cybersecurity and financial personnels. Specifically, the threat scope can be assessed based on factors including the degree of exposure of the threat attack surface, the degree of data exposure, the scope of affected assets, and the potential propagation path of the threat. The severity of consequences can be determined by assessing the degree of damage to system functions and data integrity that the threat may cause, the degree of business interruption, delay time, and recovery time. The assessment of threat potential can be determined by the success rate of the threat in attacking the organization in the past period, the number of exploitable system vulnerabilities and their CVSS scores, and the characteristics of the threat. The data can be sourced from the organization’s internal data and external cyber threat intelligence. Internal data includes past events, security logs, asset management systems, network monitoring systems, vulnerability scanning records, and other relevant information. External cyber threat intelligence includes threat intelligence analysis, industry cybersecurity reports, etc.

Threat level assessment is based on the arithmetic mean of these three sub-metrics,

14 $$TL=\raisebox{1ex}{$(TP+SC+ST)$}\!\left/\:\!\raisebox{-1ex}{$3$}\right.$$

The qualitative metrics of *potential risk (PR)* is assessed qualitatively based on the arithmetic mean of the *likelihood of future threats (LFT)* and the *severity of future threats (SFT)*. It is worth noting that in the FAIR-ROSI model, the likelihood and severity metrics under potential risks specifically assess future unpredictable threats rather than current threats.

15 $$PR=\raisebox{1ex}{$(SFT+LFT)$}\!\left/\:\!\raisebox{-1ex}{$2$}\right.$$

The specifics are as follows in Table 2.

**Table 2:** Qualitative potential risks assessment form

Likelihood of future threat	Severity of future threat	Numerical range
Highly unlikely	Minor impact	0.0–0.33
Likely	Medium impact	0.34–0.66
Highly likely	High impact	0.67–1.0

#### Baseline Setting for Quantitative Metrics

In this approach, the baseline of *ROSI* is set to 0 with the following considerations: a ROSI baseline set to 0 is the lowest investment baseline for the organization. It is in line with the principle of zero return on investment in economics because the purpose of investment is to obtain greater benefits. When the ROSI is less than 0, it means that the investment does not have any value in the analysis of the benefits of the investment. Additionally, the indicator is easy to compare and analyze. By setting the baseline to 0, it provides a greater incentive for the organization, and the members of the organization can easily achieve a ROSI greater than 0.

*Threat frequency (TF)* can be tracked and updated based on logs, monitoring systems, and other data sources. TF can be calculated using Loss Magnitude, Cost of Each Breach (CEB) and Number of Total Beaches (NTB),

16 $$TF=LM/CEB/NTB$$

The pseudocode in Sect. 4.3 shows in detail the process of how to use qualitative and quantitative metrics to evaluate ROSI through the quantitative method of the FAIR model.

#### Baseline Setting Adjustment

According to the principles of NIST Special Publication 800 − 53 Rev. 4 (FORCE, 2013), the baseline setting should be adjusted. For metrics with higher-better values, the baseline is set higher than 10% of the average historical baseline, while for metrics with lower-better values, the baseline is set lower than 10% of the average historical baseline. For example, for metrics that benefit from larger values (e.g., control measure effectiveness), the baseline is set at 110% of the metric’s average value. Conversely, for metrics that benefit from smaller values (e.g., response time and recovery time), the baseline is set at 90% of the average value. Calculating and statistically averaging values initially establishes a practical reference standard that better reflects the historical data for each organization. However, this approach considers the risks associated with overly conservative or underestimated baseline values.

## Case Study

### Case Introduction

Due to the limited accessibility of organizations context, obtaining accurate and sensitive cyber security data is challenging. Thus, this study uses a semi-synthetic data approach that combines empirical data from the IBM Cost of a Data Breach Report 2023 (IBM Security, 2023) with expert-generated data where detailed information is not available. The semi-synthetic data approach is well established in cybersecurity research and allows for comprehensive analysis while maintaining realistic parameters when detailed organizational data is not publicly available (Skopik et al., 2014). The IBM report (2023) provides insights into the cost of a data breach across industries and sizes, considering various attack vectors, the average detection and containment time for different vulnerabilities or threats, and the magnitude of impact associated with various data breaches. It also examines the financial consequences of a data breach and offers insights into the factors influencing these costs. The case hypotheses in this paper will be based on specific data from the report, which includes total loss cost, data breach cost based on organization size, and threat response and recovery time averages. For metrics not directly provided in the report, we supplemented expert-generated data with inputs from three cybersecurity practitioners, each with more than 10 years of experience in enterprise security operations and risk management.

Companies A and B are both hypothetical companies based on real data from the IBM 2023 data breach report. Company A is a professional cyber security service provider, and Company B is a new e-commerce platform with a workforce of 10,000 to 25,000 employees. Due to the rapid expansion of Company B’s business in recent years, it has experienced an increase in phishing incidents, leading to substantial user data leakage. It has resulted in business interruptions, affecting the organization’s normal operations, and causing significant losses.

### Case Statistics

The primary challenge faced by Company B is the substantial data leakage of customer, employee, and anonymous data due to numerous phishing incidents. The statistics reveal that the total cost of losses amounts to $5,360,000, corresponding to the loss magnitude (LM). The main data leakage stems from a significant number of phishing incidents, accounting for $5,360,000. Among these, the leakage of customer information constitutes a major portion, with each data leakage incident costing $183.

Company B then implemented a range of security measures, including Intrusion Detection Systems (IDS), Intrusion Prevention Systems (IPS), and Multi-Factor Authentication (MFA), as well as the incorporation of security artificial intelligence and automated detection. Following the implementation of these control measures, the new loss magnitude (NLM) is $2,650,000. During a specific period for statistics on security metrics, the following results are obtained: a response time of 167 days, a recovery time of 47 days, security configuration compliance rate measures at 97%, vulnerability patching rate is at 88% and attack block rate at 99%. The threat likelihood metric is recorded at 0.4, the threat consequence severity is 0.6, and the threat impact range is 0.65. The potential risk likelihood metric is measured at 0.33, and the potential risk consequence severity is 0.6. Additionally, the threat frequency is found to be 27 times per year.

In this context, Company A needs to assess the situation, compares it with the baseline and advise on the return on the cyber security investment.

### Baseline Setting

#### Setting Qualitative Baseline

According to the Cost of a Data Breach Report 2023 (IBM Security, 2023), IBM conducted statistical analysis to determine the averages of the two sub-metrics within the qualitative metrics for *threat response capability*. These are an average *response time* of 217 days and an average *recovery time* of 76 days. Furthermore, it’s assumed that the current organization’s *security configuration compliance* rate is 84%, *vulnerability patching rate* is 78% and *attack block rate* is 92%. As for the *threat level*, we assume the average values, derived from statistical analysis and assessment, for the organization’s *threat potential*, *severity of consequences*, and *scope of threat* to be 0.5, 0.8, and 0.8, respectively. For *potential risks*, we assume that the average value of the *likelihood* of potential risk values in the historical data of the organization is 0.4, while the average value of the *severity* of potential risk consequences is 0.8. According to the principles outlined in 3.3.1 and 3.3.3 (i.e., the 10% baseline setting adjustment), the following baseline metrics for the organization’s existing threat response capability, threat level and potential risks can be established in Table 3.

**Table 3:** Mean value and baseline of qualitative and quantitative indicators

Cybersecurity Metrics	Sub-metrics		Mean Value	Baseline
Threat response capability	Pre-incident control effectiveness	Security configuration compliance rate	84%	84$\:\times\:$(1$\:+$10%) = 92.4
		Vulnerability patching rate	78%	$\:78\times\:$(1$\:+$10%) = 85.8
		Attack blocking rate	92%	92$\:\times\:$(1$\:+$10%) =101.2
	Post-incident capability	Response time (days)	217	217$\:\times\:$(1$\:-$10%) = 195
		Recovery time (days)	76	76$\:\times\:$(1$\:-$10%) = 68
Threat level	Threat potential		0.5	0.5$\:\times\:$(1$\:-$10%) = 0.45
	Severity of consequence		0.8	0.8$\:\times\:$(1$\:-$10%) = 0.72
	Scope of threat		0.8	0.8$\:\times\:$(1$\:-$10%) = 0.72
Potential risks	Likelihood of future threats		0.4	0.4$\:\times\:$(1$\:-$10%) = 0.36
	Severity of future threats		0.8	0.8$\:\times\:$(1$\:-$10%) = 0.72
Threat frequency	Threat frequency (times/year)		37	33
ROSI	ROSI		n.a.	0

#### Setting Quantitative Baseline

As mentioned in Sect. 3.3.2, the baseline of *ROSI* is set to 0. It is in line with the principle of zero return on investment in economics because the purpose of investment is to obtain greater benefits. When the *ROSI* is less than 0, it means that the investment does not have any value in the analysis of the benefits of the investment.

 As for the threat frequency, from the report, the cost of data leakage is $5,360,000 per year. The main data breaches involve customer information leakage problems, and the cost of each data breach is $183. In this regard, it is estimated that the number of data breaches caused by the number of data breaches per year is 5,360,000/183 = 29,290. In response, there are a total of 29,290 data breaches per year. Thus, considering the size of the organization and the cost of a data breach, and if each threat event (e.g., phishing event) results in an average of about 800 data breaches, the annual threat frequency is 29,290/800 = 37. According to the 10% baseline adjustment, the organization’s threat frequency baseline is adjusted to 33 (Table 3 ). Table 3 presents the data means of each metric in the study.

### Use FAIR-ROSI Model To Analyze Return on Security Investment

Considering the statistics from the report (see Sect. 4.2) as inputs as well as reasonable assumptions, we calculated the return on investment after the organization implemented security measures. The specific analysis process is outlined in the following algorithm.


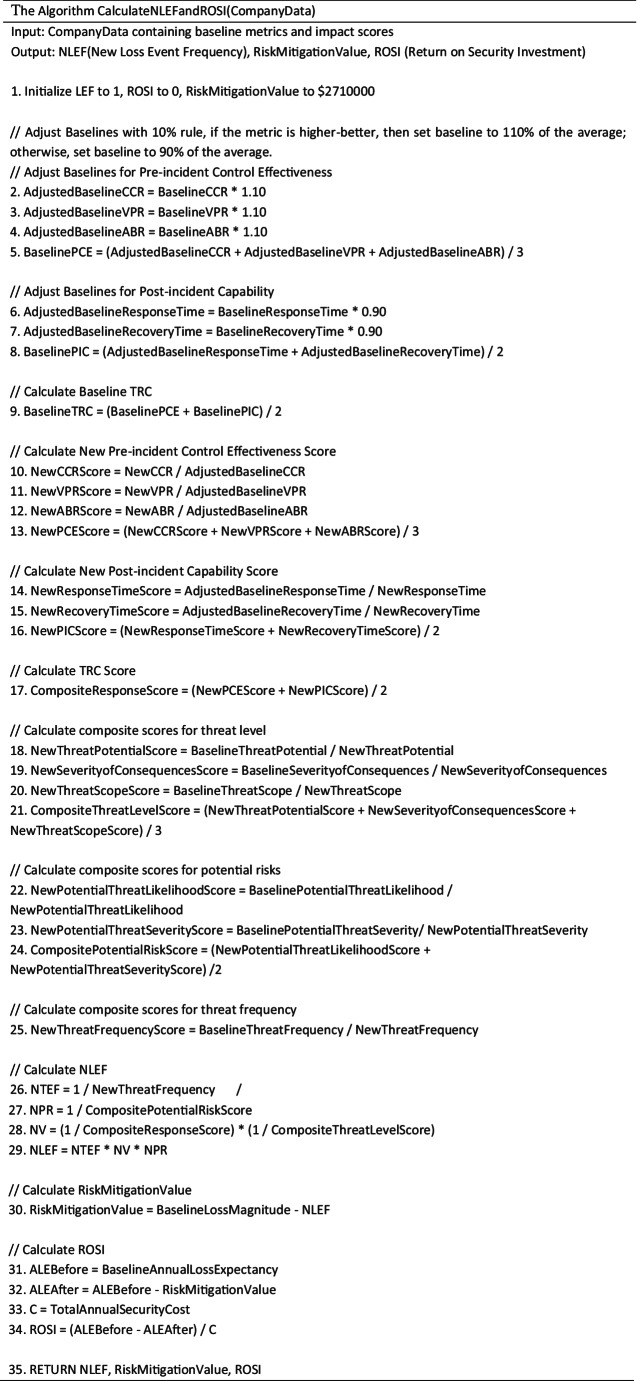


The analysis process includes the calculation of composite scores for threat response capability, composite scores for threat severity, composite scores for potential risk, risk mitigation value and ROSI. We aim to compare the loss before and after applying the new security measures which is detailed in Sect. 4.5.

### Results Analysis

 This section presents the comparison of losses before and after implementing the security measures, as shown in Table 4. Based on the results of IBM Security ( 2023 ) actual statistics, Company B reduced the actual loss margin (the actual value at risk) from $5,360,000 to $2,650,000 by implementing a series of security measures. When using the research method and research metrics identified in this paper, the calculated expected risk loss value was $2,551,701 per year. A comparison revealed that the actual risk loss value is closer to the expected risk loss value. The expected risk loss value is relatively close. Additionally, when comparing with Company B’s costs for detection and notification upgrades totaling $1,950,000 per year, it becomes evident that the degree of risk mitigation (i.e., the reduction in risk value) significantly exceeds the costs incurred by Company B.

**Table 4:** Comparison of scores for qualitative and quantitative indicators before and after implementing security measures

Cybersecurity metrics	Metrics		Before implementation of security measures (Company B baseline)	After implementation of security measures
Threat response capability	Composite score		1	1.21
	Pre-incident control effectiveness	Composite score	1	1.018
		Security configuration compliance rate	92.4	97
		Vulnerability patching rate	85.8	88
		Attack blocking rate	101.2	99
	Post-incident capability	Composite score	1	1.307
		Response time (days)	195	167
		Recovery time (days)	68	47
Threat level	Composite score		1	1.144
	Threat potential		0.45	0.4
	Severity of consequence		0.72	0.6
	Scope of threat		0.72	0.65
Potential risks	Composite score		1	1.145
	Likelihood of future threats		0.36	0.33
	Severity of future threats		0.72	0.6
Threat frequency	Composite score		1	1.22
	Threat frequency (times/year)		33	27
Other	ROSI		0	1.39
	Actual loss value		$5,360,000	$2,650,000

Furthermore, to better visualize the benefits of this investment, this paper uses the risk mitigation value to calculate the ROSI, resulting in a ROSI calculation of 1.39. A comparison indicates that this value (1.39) is significantly greater than the baseline ROSI of 0, which signifies a very high benefit from the investment. Naturally, before investing, Company B can establish its risk tolerance level according to its specific circumstances and organizational fundamentals. The company may choose not to implement the security measures, if the calculated risk value remains below the established threshold.

## Discussion

This study developed a comprehensive cybersecurity investment assessment model, which for the first time attempts to integrate the FAIR model and the ROSI model. We mapped the qualitative and quantitative indicators related to cyber threats (e.g., threat level, threat frequency, threat response capability) to the relevant components of the FAIR model. By using the risk quantification framework and calculation method of the FAIR model, the FAIR-ROSI model can calculate the ROSI based on the values of the indicators and assess the economic impact of cybersecurity measures. The model is further demonstrated through a case study that uses data from the 2023 IBM Data Breach Cost Report to assess the financial return of a specific cybersecurity investment. This study makes contributions to both the theoretical and practical aspects of cybersecurity investment.

### Theoretical Contribution

The FAIR-ROSI model enriches the methodological framework for cybersecurity investment decisions and makes two major contributions to promoting responsible cybersecurity investment by improving the accuracy, comprehensiveness, and transparency of investment decisions.

On one hand, this study integrates the FAIR model with the ROSI model in a novel way, using the FAIR model to quantify risk and incorporate it into the ROSI model, integrating both quantitative and qualitative analysis. This provides organizations with more accurate and comprehensive risk assessment and cost-benefit analysis. Specifically, this approach connects the value of risk with both Loss Event Frequency (LEF) and Loss Magnitude (LM). It fully addresses the multiple variables impacting LEF, including Threat Event Frequency (TEF) and Vulnerability (V) and captures the maximal influence that these factors exert on the value of risk. Through the use of the FAIR model to quantify a plethora of qualitative and quantitative metrics, it amends and enhances the LEF and offers a precise basis for organizational risk assessment. Built on both quantitative and qualitative metrics, the FAIR component of the FAIR-ROSI model delivers precise risk values, while the ROSI component integrates economic considerations. This analysis empowers organizations to gain a comprehensive understanding of the impact of diverse threats and cyber risks on their organization. Furthermore, the FAIR model provides quantitative risk scenarios, and the ROSI model measures the relationship between risk mitigation values and costs. This paper’s fusion of the FAIR and ROSI models serves not only to quantify risk and assess the effectiveness of risk mitigation but also to merge risk management with cost-effectiveness. The result is a more holistic, precise, and targeted risk management analysis within organizations. This combination facilitates a superior grasp and measurement of risk that enables informed decision-making and optimal risk management strategies, particularly when dealing with limited resources.

On the other hand, by mapping technical cybersecurity metrics to the FAIR model, we translate technical terms to financial terms that can be understood by both the CISO, and other members of the decision-making board who often lack in-depth knowledge of their system architecture, the threats faced, and the existing vulnerabilities (Moore et al., 2016). Current cybersecurity investment decision-making heavily relies on the CISO’s experiential judgment (Rowe & Gallaher, 2006). However, this process is opaque like a “black box”, which is subject to the risk of subjective bias (Borrero & Henao, 2017; Busenitz & Barney, 1997). Traditional models such as ROSI as an economic indicator can be used to assess investment efficiency (Garvey et al., 2013; Gordon et al., 2020), however, it focuses solely on pure financial results (Kesswani & Kumar 2015), lacking in specific guidance on how to translate the risks posed by cyber threats into financial losses or costs. Existing research have also attempted to integrate economic and risk factors into comprehensive frameworks. For example, Wang et al. (2020) designed a more complex risk analysis model by combining Bayesian networks and FAIR model, which quantifies cybersecurity risks by categorizing them into measurable components such as Loss Event Frequency (LEF) influenced by Threat Event Frequency (TEF) and vulnerability. Nagurney et al. (2017) constructed a multi-objective optimization model in the supply chain network to minimize security costs and maximize risk reduction. However, existing hybrid models failed to bridge the gap between technical security indicators and financial decisions, and the complexity of the model itself leads to the opacity of its output, which hinders the decision makers’ trust.

Our proposed FAIR-ROSI model maps cybersecurity metrics from the technical domain to the corresponding components of the FAIR model and develops a calculation method to incorporate them into the ROSI assessment process. Specifically, we used the Threat Frequency (TF) instead of the Threat Event Frequency (TEF) as it is a more intuitive and accessible metric. Unlike the TEF, TF is easier to derive from historical data and system logs, making it more concrete and operational. This substitution improves the model’s intuitiveness, operability, and data availability. The choice of mapping Threat Responsiveness (TR) to Resistance Strength (RS) is based on the high degree of quantifiability of TR. Threat responsiveness can typically be measured using specific metrics, such as response time, recovery time, and the effectiveness of control measures. These are quantifiable data points that can be more easily correlated with RS, which offers a way to quantify security performance. Furthermore, mapping Threat Level (TL) to Threat Capability (TC) is justified because TL is a measure of the severity of a threat, describing its impact on the organization. By mapping TL to TC, the model’s accuracy can be improved as it reflects the potential threats’ impact on the organization. Moreover, the impact of threat level on the organization can be quantified by quantifying the threat likelihood, threat consequence severity, and threat impact range within the threat level. Therefore, the choice of threat level to map to TC takes into consideration the comprehensiveness of the metrics. Additionally, this research introduces Potential Risk (PR) to modify the Loss Event Frequency (LEF), which improves the model’s foresight and predictive capabilities. This unique theoretical approach directly embeds key factors that IT experts pay attention to in cyber risk assessment into the specific framework of the financial risk model, which to a certain extent opens the “black box” of cybersecurity investment decision evaluation, improves the transparency of the cybersecurity investment decision process, and effectively responds to existing challenges.

### Practical Implication

From a practical perspective, the FAIR-ROSI model provides a transparent assessment method for cybersecurity investments. It is particularly helpful for small and medium-sized enterprises (SMEs) to make cybersecurity investment decisions as SMEs typically face tight budgets and limited cybersecurity resources (Alahmari & Duncan, 2021; Heidt et al., 2019). Their decision-makers are often not experts in the field of cybersecurity and may lack the necessary technical knowledge and experience to make complex cybersecurity investment decisions (Miaoui & Boudriga, 2019). The FAIR-ROSI model provides them with a transparent framework that translates complex cybersecurity risks into measurable financial metrics. The inherent objectivity of some metrics used in the FAIR-ROSI model reduces the reliance on the CISO’s subjective judgement and experience in investment assessment process, allowing organizations to understand the causes and effects of the ROSI metric clearly and make responsible investment decisions. For example, if an organization has a robust logging and monitoring system, some sub-metrics in the FAIR-ROSI model such as threat incident response time, threat incident recovery time, and threat frequency can be obtained through network and system monitoring tools, manual event reporting, or automated event logging. Additionally, clearly defining the assessment mechanisms for other qualitative metrics (e.g., threat level, potential risks) can help other decision-makers in the organization understand the reasoning behind the CISO’s cybersecurity risk assessments.

Furthermore, the case study in this research demonstrates the feasibility of the model to real-world scenarios and offers practical insights. As we can see, after the implementation of control measures in Company B, it can be observed that the three qualitative metrics have improved, and their composite scores are all greater than the baseline score of 1. Furthermore, the quantitative metrics, specifically threat frequency, have also shown improvement. Upon comparing the expected risk value and the actual risk value, it is evident that the two values are relatively close. This indicates that the selected metrics and the mapping of metrics to the LEF are highly scientifically reasonable, and the use of these metrics accurately reflects the cybersecurity situation of Company B.

Finally, the FAIR-ROSI model also support organizations to fulfill their ethical and regulatory obligations. As organizations embrace digital transformation, they need to protect sensitive data and ensure the security of their digital infrastructure (Fleischman et al., 2023). FAIR-ROSI model provides a robust cybersecurity investment assessment framework based on transparent and quantifiable cybersecurity metrics, which enables organizations to identify their cybersecurity weaknesses more accurately, allocate resources reasonably, ensure the sustainability of cybersecurity investments and offer security guarantees for their digital transformation. By doing this, organizations can not only perform commitments to customers, employees, and other stakeholders, handle shared data and information safely, compliantly, and responsibly, but also achieve regulatory compliance requirements and social responsibilities in cybersecurity.

### Limitation and Future Work

While the FAIR-ROSI model offers valuable insights, there are still several limitations. One limitation is that the selected metrics and their sub-metrics do not cover an exhaustive list of factors relevant to quantifying cyber risk. However, our model focuses on improving the transparency of the qualitative metrics assessment through using quantifiable sub-metrics (e.g., vulnerability patching rate, recovery time). We also identified new metrics including quantifiable factors such as the degree of exposure of the threat attack surface, the potential propagation path of the threat. These metrics can be assessed and determined using multiple data sources: operational data (e.g., security logs, SIEM alerts), infrastructure data (e.g., asset management systems, network monitoring systems), threat intelligence feeds and experts’ input. There may be other metrics that can provide more insights, which requires multiple in-depth industrial case studies to explore further and validate these metrics. Future research can build on this research to develop more comprehensive and specialized metrics suitable for different industries.

Furthermore, our model treats the relationships between metrics at different levels of abstraction (e.g., recovery time vs. threat potential) as linear and additive. The model can be further improved to capture more complex interactions between these metrics. In addition, the effectiveness of the model also depends on the quality and completeness of the input data. With the advancements in cyber threat intelligence and its products, this should not be a concern as the quality of CTI data continues to improve.

Another constraint is that it is applied to a case study derived from the IBM 2023 Data Breach Report (IBM Security, 2023), and not to an actual organization with real-life data. Applying the model to empirical data from an actual case study would greatly strengthen its validity and enhance its practical contribution. This step would allow for a more nuanced understanding and validation of the model in a practical context. However, the extensive and complex nature of conducting such an empirical study necessitated its exclusion from this current research phase. The next phase of our research will focus on a detailed exploration and analysis of the model using empirical data and real-world cases, aligning with the rigorous standards required for a thorough empirical investigation. This future work allows us to validate and refine our model in real world practice.

## Conclusion

This paper marks the first effort to merge the FAIR and ROSI models, quantifying and comparing expected and actual risk values in an organization. It improves the understanding of metric validity and baseline settings and explores risk mitigation and ROSI’s impact on investment decisions. This approach offers a more scientific method for risk estimation and analyzing investment returns. However, the study acknowledges limitations, and future work will aim to apply the framework to real cases and refine the metrics and models to offer a comprehensive and current cybersecurity decision support guide. This FAIR-ROSI model represents a significant advancement in protecting user data, mitigating financial disruptions, maintaining the integrity of digital infrastructure. Ongoing and sustainable cyber security investments also reflect organization’s commitment to social responsibility, as organizations comply with local and international policies and regulations, and fulfil their responsibilities to users, partners, and society as responsible participants in the digital economy.

## Data Availability

The relevant data are available from the corresponding author upon request.
